# The Molecular Roles and Clinical Implications of Non-Coding RNAs in Gastric Cancer

**DOI:** 10.3389/fcell.2021.802745

**Published:** 2021-12-13

**Authors:** Yanping Yue, Xinrong Lin, Xinyue Qiu, Lei Yang, Rui Wang

**Affiliations:** ^1^ Department of Medical Oncology, Affiliated Cancer Hospital, Nantong University, Nantong, China; ^2^ Department of Medical Oncology, Affiliated Jinling Hospital, Medical School of Nanjing University, Nanjing, China

**Keywords:** gastric cancer, non-coding RNA, therapy resistance, microRNA, lncRNA

## Abstract

Gastric cancer (GC) is one of the most common malignancies in the world. It is also the fifth most common cancer in China. In recent years, a large number of studies have proved that non-coding RNAs (ncRNAs) can regulate cell proliferation, invasion, metastasis, apoptosis, and angiogenesis. NcRNAs also influence the therapeutic resistance of gastric cancer. NcRNAs mainly consist of miRNAs, lncRNAs and circRNAs. In this paper, we summarized ncRNAs as biomarkers and therapeutic targets for gastric cancer, and also reviewed their role in clinical trials and diagnosis. We sum up different ncRNAs and related moleculars and signaling pathway in gastric cancer, like Bcl-2, PTEN, Wnt signaling. In addition, the potential clinical application of ncRNAs in overcoming chemotherapy and radiotherapy resistance in GC in the future were also focused on.

## 1 Introduction

Gastric cancer (GC) is the fifth most common type of cancer and the third most common cause of cancer-related deaths worldwide after lung and colorectal cancer (Bray et al., 2018). In 2018, the global age-standardized incidence and mortality rates for GC were 11.1 and 8.2 per 100,000 persons, respectively (Thrift and El-Serag, 2020). The Global Burden of Disease Study (GBD) 2017 showed that the global age-standardized incidence decreased by 28.0% in 2017 compared with that in 1990, and the age-standardized mortality decreased by 48.7% (ollaborators, 2020). The uniform declines in incidence have been observed in many parts of the world for decades, including China (Luo et al., 2017). Early detection of gastric cancer is considered to have contributed to favorable survival (Sekiguchi et al., 2021). In 2004, an early detection and treatment program was initiated in China with special funds from the Ministry of Health (Zheng et al., 2015). Therefore, one of the main reasons for the improvement of gastric cancer is the popularity of endoscopic screening (Zhang et al., 2018a). Endoscopic resection is the preferred treatment for early GC, whereas traditional surgery, including D2 lymphadenectomy, consisting of lymph node stations in the perigastric mesentery and along the celiac arterial branches, is the main treatment for operable GC (Hiki et al., 2013; Pimentel-Nunes et al., 2015). The first line of treatment for patients with advanced GC consists of sequential chemotherapy with platinum and fluoropyrimidine (Smyth et al., 2016). Targeted therapies currently approved to treat GC include trastuzumab as first line therapy for HER2-positive patients, ramucirumab as an anti-angiogenic agent for second line treatment, and nivolumab or pembrolizumab as an anti-PD-1 agent for third line treatment (Group et al., 2010; Fuchs et al., 2014; Kang et al., 2017). Some patients, however, have multiple drug resistance (MDR) to these agents, leading to a poor prognosis. Understanding the mechanisms underlying resistance to these drugs is needed to develop new methods for accurate early detection and effective treatment of GC.

**FIGURE 1:**
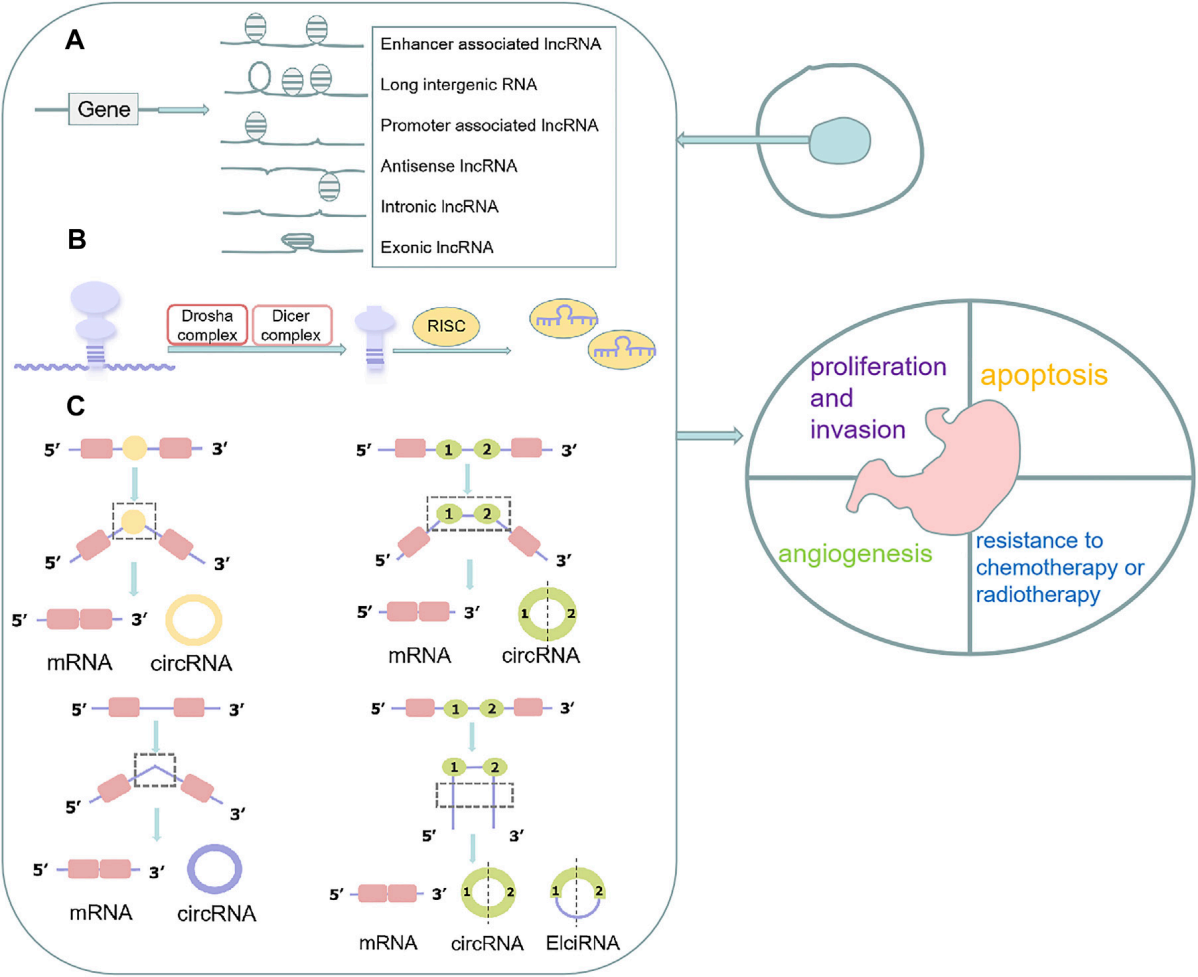
Function, biogenesis and localization of ncRNAs in cells. **(A)** lncRNAs are classified into various categories, including promoter-related lncRNAs, enhancer-related lncRNAs, intronic and exonic lncRNAs, antisense lncRNAs, and long intergenic lncRNAs; **(B)** The pre-miRNAs are loaded into the RNA-induced silencing complex (RISC) where the mature single-stranded miRNA binds to complementary mRNA targets after processed by Drosha complex and Dicer complex; **(C)** CircRNAs are classified into different types, including intron circRNAs, exon circRNAs, and extron-intron circRNAs.

Non-coding RNAs (ncRNAs) are unique transcripts that do not encode proteins (Esteller and Pandolfi, 2017). According to the length and shape, ncRNAs can be subdivided into the following types: tiny/short ncRNAs, long ncRNAs (lncRNAs) and circular RNA (circRNAs). There are various small ncRNAs, such as microRNAs (miRNAs), PIWI-interacting RNAs (piRNAs), small nucleolar RNAs (snoRNAs) and small nuclear RNAs (snRNAs) (Wei et al., 2019). The most widely studied class of ncRNAs are miRNAs, which are small ncRNAs of ∼22 nucleotides (nt) that, in animals, mediate post-transcriptional gene silencing by controlling the translation of mRNA into proteins (He and Hannon, 2004). LncRNAs are a heterogeneous group of non-coding transcripts more than 200 nt long that are involved in many biological processes (Esteller, 2011). PiRNAs are ncRNAs of 24–30 nt in length, are Dicer-independent and bind the PIWI subfamily of Argonaute family proteins that are involved in maintaining genome stability in germline cells (Aravin et al., 2007). SnoRNAs are intermediate-sized ncRNAs (60–300 bp). They are components of small nucleolar ribonucleoproteins (snoRNPs), which are complexes that are responsible for sequence-specific 2′-O-methylation and pseudouridylation of ribosomal RNA (rRNA) (Ni et al., 1997). Several of these ncRNAs have been found to act as key regulators of various cell functions in GC, including cell proliferation, apoptosis, the cell cycle, and cellular metabolism (Chun-Zhi et al., 2010; Liu et al., 2019a; Zhang et al., 2019a). Moreover, some ncRNAs were reported closely related to the development of chemoresistance in GCs (Liu et al., 2019a; Xi et al., 2019) (Figure 1).

This review systematically summarizes current knowledge about the mechanisms by which ncRNAs regulate cell proliferation, invasion, and apoptosis, as well as their impact on drug resistance. Elucidation of these mechanisms may provide insight into the future use of ncRNAs as hypothetical biomarkers and/or therapeutic targets of GC.

## 2 Mechanisms of ncRNAs That Regulate Gastric Cancer Behavior

Multiple ncRNAs dysregulate the behavior of GC by post-transcriptionally binding to the 3′UTR of downstream gene mRNAs, including mRNAs encoded by various oncogenes, such as *EZH2*, *EGFR*, *STRA6*, *FSCN1*, *TRIM24*, *PDK4*, *LYN*, and *PPP2R2A*, and suppressor genes, such as *PTEN*, *RUNX1*, and *YPEL1* (Table 1) (Markopoulos et al., 2017). In addition, complex interactions among internal members play crucial roles in proliferation, apoptosis, angiogenesis, and other behaviors by affecting classic signaling pathways.

**TABLE 1:** NcRNAs and oncogenes and suppressor genes in gastric cancer behavior.

NcRNA	Targeted genes	Genetic properties	References	behavior
lncRNA HOXA11-AS;lncRNA MNX1-AS1;lncRNA FOXP4-AS1	EZH2	oncogene	Liu et al. (2017); Shuai et al. (2020); Chen et al. (2019a)	proliferation
lncRNA LINC00673;lncRNA FOXD2-AS1;lncRNA UCA1			Huang et al. (2017a); Xu et al. (2018); Wang et al. (2017)	
lncRNA SNHG6;lncRNA SPRY4-IT1;lncRNA PART1			Li et al. (2018a); Zhou et al. (2017a);Han et al. (2020)	
lncRNA AFAP1-AS1;lncRNA SNHG17;lncRNA AGAP2-AS1			Yuan et al. (2020); Zhang et al. (2019b); Qi et al. (2017a)	
lncRNA OIP5-AS1;lncRNA CASC15;lncRNA HOTAIR			Bai and Li (2020); Wu et al. (2018a); Song et al. (2019)	
miR-101;miR-31;miR-26b			Carvalho et al. (2012); Sun et al. (2019); Rossi et al. (2019)	
miR-370;miR-138-5p;miR-204-5p;miR-146a	EGFR	oncogene	Ning et al. (2017); Wang et al. (2018a); Kogo et al. (2011)	
miR-873	STRA6	oncogene	Lin et al. (2019)	
miR-133b	FSCN1	oncogene	Guo et al. (2014)	
miR-511	TRIM24	oncogene	Fang et al. (2017)	
miR-216a;miR-18a	RUNX1	suppressor gene	Wu et al. (2018b)	
			Qi et al. (2020)	
miR-5683	PDK4	oncogene	Miao et al. (2020)	
miR-885	YPEL1	suppressor gene	Li et al. (2019a)	
miR-122	LYN	oncogene	Meng et al. (2020)	
miR-665	PPP2R2A	oncogene	Zhang et al. (2020a)	
LINC00152;miR-383;miR-1915-3p;miR-24	Bcl-2	oncogene	Tao et al. (2021); Cui et al. (2019); Mao et al. (2019); Zhang et al. (2016a)	apoptosis
miR-23a/b;miR-499-5p;miR-183; miR-93	PDCD4	suppressor gene	Hu et al. (2017); Yang et al. (2020); Li et al. (2016); Liang et al. (2016)	
miR-200a;lncRNA XIAP-AS1	TRAIL	suppressor gene	Guo et al. (2018); Cai et al. (2017)	
LncRNA PVT1;miR-125a;miR-1	VEGF	oncogene	Dai et al. (2015); Xie et al. (2018); Zhao et al. (2018); Xie et al. (2020a)	angiogenesis
X26nt;lncRNA MALAT1	VE-cadherin	oncogene	Chen et al. (2021a); Li et al. (2017a)	

### 2.1 Proliferation and Invasion of ncRNAs

*EZH2*, which plays a central role in all aspects of GC pathogenesis, is more highly expressed in GC tissues than in non-tumor epithelium, with increased expression associated with more aggressive biological behavior and poor prognosis of GC (Gan et al., 2018). The lncRNAs HOXA11-AS, MNX1-AS1, and FOXP4-AS1, and the microRNAs -101, -31, and -26b, all have an impact on the progression of GC by altering the level of expression of *EZH2* (Carvalho et al., 2012; Huang et al., 2017a; Qi et al., 2017a; Zhou et al., 2017a; Liu et al., 2017; Wang et al., 2017; Li et al., 2018a; Wu et al., 2018a; Xu et al., 2018; Chen et al., 2019a; Zhang et al., 2019b; Rossi et al., 2019; Song et al., 2019; Sun et al., 2019; Bai and Li, 2020; Han et al., 2020; Shuai et al., 2020; Yuan et al., 2020). Epidermal growth factor receptor (EGFR) is an oncogenic transmembrane receptor that is overexpressed in many cancers, including GC (Zhen et al., 2014). MiRs-370 (Ning et al., 2017), -138-5p, and -204-5p (Wang et al., 2018a), and -146a (Kogo et al., 2011) target EGFR, thereby altering the migration and proliferation of GC cells. MiR-873, which down-regulates *STRA6* mRNA in GC, plays a suppressor role (Lin et al., 2019). MiR-133b directly targets and inhibits *FSCN1*, which acts as an oncogene in GC cells, whereas miR-511 inhibits the tumor suppressor gene *TRIM24* in GC (Guo et al., 2014; Fang et al., 2017). MiR-216a and miR-18a directly target and downregulate *RUNX1* (Wu et al., 2018b; Qi et al., 2020); and miR-5683 promotes glycolysis and progression by up-regulating *PDK4* expression (Miao et al., 2020). In addition, miR-885, which targets *YPEL1*, enhances GC cell proliferation, colony formation, and invasion; and miR-122 and miR-665 inhibit proliferation, invasion, and EMT by reducing the expression of *LYN* and *PPP2R2A*, respectively, in GC (Li et al., 2019a; Zhang et al., 2020a; Meng et al., 2020).

In addition, many other lncRNAs and circRNAs act as competitive endogenous RNAs (ceRNAs) of miRNAs, regulating the expression of oncogenes or tumor suppressor genes in GC. For example, the lncRNA LINC0130, which inhibits miR-101-3p activity, enhances the expression of *EZH2*, thereby promoting GC progression (Cao et al., 2019). The lncRNA MT1JP has been shown to act as a ceRNA for miR-92a-3p, up-regulating the expression of *FBXW7* and reducing GC cell proliferation (Zhang et al., 2018b). The lncRNA LINC00240 promotes GC through the LINC00240/miR-124-3p/DNMT3B axis as an oncogene (Li et al., 2020a). By binding to and neutralizing miR-286-5p, CircPSMC3 enhances the expression of *PTEN* (Rong et al., 2019). CircGRAMD1B plays a negative role in GC progression by affecting the miR-130A-3P-PTEN/P21 axis (Dai et al., 2019). CircFAT1 binds to and neutralizes miR-548g, increasing the expression of the tumor suppressor gene *RUNX1* in GC cells (Fang et al., 2019). Hsa-circ-0017639 promotes GC proliferation and metastasis by binding to and neutralizing miR-224-5p, thereby up-regulating *USP3* expression (Li et al., 2020b).

NcRNAs are involved in many pathways, including the PTEN/PI3K/AKT/mTOR, Hippo, and Wnt/β-catenin signaling pathways, adding a new dimension to the understanding of GC progression. The LncRNA BX357664, which acts as a ceRNA of miR-183-3p to target *PTEN*, affects the PI3K/AKT/mTOR pathway and inhibits GC proliferation and metastasis (Liang et al., 2021). LncRNA HORAIRM1 acts as a ceRNA of miR-17-5p to up-regulate the expression of *PTEN* and mediate the PI3K/AKT pathway (Lu et al., 2019). By binding to and neutralizing miR-149-5p, circNRIP1 affects the level of expression of the AKT/mTOR axis and acts as a tumor promoter in GC (Zhang et al., 2019c). Overexpression of the circRNA ciRS-7 inhibits the tumor suppressor effect of miR-7 through the PTEN/PI3K/AKT signaling pathway (Pan et al., 2018). The lncRNA HCG18, which inhibits the expression of miR-141-3p; the lncRNA LINC00649, which targets miR-16-5p; and circLARP4, which adsorbs miR-424 are all directly implicated in the proliferation and invasion of GC cells by influencing the Hippo signaling pathway (Zhang et al., 2017a; Liu et al., 2020). The lncRNAs LINC01133, OIP5-AS1, and LINC01355, which bind to and neutralize miR-106a-3p, miR-367-3p, and miR-431-5p, respectively, all activate the Wnt/β-catenin signaling pathway and induce cell proliferation (Yang et al., 2018a; Tao et al., 2020; Piao et al., 2021).

### 2.2 Apoptosis of ncRNAs

At present, the known apoptosis-related genes in GC include those encoding the anti-apoptosis-related factors genes B-cell lymphoma-2 (Bcl-2), programmed cell death 4 (PDCD4), and tumor necrosis factor-alpha-related apoptosis-inducing ligand (TRAIL) (Carthy et al., 2003; Wu et al., 2004; Wang et al., 2010; Zhu et al., 2016; Lim et al., 2017). In addition, gastric cancer cells may undergo apoptosis through the MAPK/ERK, PI3K/Akt/mTOR, Wnt/β-catenin and other signaling pathways (Fattahi et al., 2020; Su et al., 2020). Many studies have found that ncRNAs related to these genes and signaling pathways play important roles in the GC cell apoptosis.

The anti-apoptotic protein Bcl-2 has been shown to play an important role in GC. MiR-383 and miR-1915-3p have been shown to reduce Bcl-2 expression in GC, whereas LINC00152 has the opposite effect (Cui et al., 2019; Mao et al., 2019; Tao et al., 2021). The expression of BCL2L11, a pro-apoptotic member of the Bcl-2 family, is reduced by miR-24, thereby inhibiting apoptosis (Zhang et al., 2016a). The roles of tumor suppressor PDCD4 in GC mainly include promoting cell apoptosis. PDCD4 may be a key downstream protein in the development of GC (Zhao et al., 2020). MiR-23a/b, miR-499-5p, miR-183, miR-93 all target and negatively regulate PDCD4 to promote GC development (Li et al., 2016; Liang et al., 2016; Hu et al., 2017; Yang et al., 2020). TRAIL is a protein that promotes apoptosis in cancer cells by inducing the formation of the death-inducing signal complex (DISC). Over-expression of miR-200a and knockdown of the lncRNA XIAP-AS1 enhance TRAIL-induced apoptosis in GC cells (Cai et al., 2017; Guo et al., 2018).

MiR-206 targets and suppresses *MAPK3* mRNA, whereas miR-135b activates MAPK/ERK signaling, thereby affecting GC cell apoptosis (Chen et al., 2019b; Zhou and Chen, 2019). The lncRNA BCAR4 facilitates MAPK/ERK signaling, whereas the lncRNA LINC00858 reduces the methylation of the WNK2 gene promoter and its downstream MAPK signaling pathway (Zhou et al., 2020; Du et al., 2021). As such, ncRNAs function as master regulators of apoptosis in GC by controlling two other signaling pathways, the PI3K/Akt/mTOR and Wnt/β-catenin pathways. Some of these regulatory molecules, including miR-495 (Wang et al., 2018b), miR-193b (Tian et al., 2020a), miR-139-5p (Zhang et al., 2020b), lncRNA SLC25A5-AS1 (Li et al., 2019b), and miR-195-5p (Zhao and Wu, 2019), inactivate both pathways; whereas others, including miR-194 (Peng et al., 2018), miR-324-3p (Sun et al., 2018), and LINC00355 (Luan et al., 2020), activate both pathways to inhibit apoptosis of GC cells.

### 2.3 Angiogenesis of ncRNAs

Tumor expansion depends on angiogenesis, as blood vessels provide oxygen and nutrients to tumors (Carmeliet and Jain, 2011). Angiogenesis is therefore essential for tumor occurrence, progression, invasion, migration, and metastasis (Hanahan and Weinberg, 2000). Tumors and stromal cells secrete abnormal levels of growth factors that cause tumor vascular dysplasia, including vascular disorders, immaturity, and permeability (Viallard and Larrivée, 2017). The important role of angiogenesis in gastric cancer and other tumors has been extensively studied. Inhibition of the angiogenesis signaling pathway will help prevent tumor growth and prolong the survival time of cancer patients. For gastric cancer, the REGARD trial, RAINBOW trial, and several ongoing RCTs have shown that ramucirumab that targeting VEGF signals help GC patients to achieve better survival (Li et al., 2021).

Continuous expression of vascular endothelial growth factor (VEGF) can stimulate angiogenesis (Maeda et al., 1996). Low expression of miR-125a maintains the secretion of VEGF-A in GC, thereby modulating tumor angiogenesis (Dai et al., 2015). Overexpression of miR-1 inhibits the growth of blood vessels by reducing the expression of VEGF-A and endothelin 1 (EDN1) (Xie et al., 2018). The lncRNA PVT1 plays an important role in GC angiogenesis by activating the STAT3/VEGFA axis (Zhao et al., 2018). The adsorption of circSHKBP1 on exosomes by miR-582-3p enhances the stability of VEGF mRNA (Xie et al., 2020a).

Vascular endothelial cadherin (VE-cadherin) has been shown to enhance vascular permeability, angiogenesis and tumor growth (Viallard and Larrivée, 2017). The exosomal 26‐nt‐long ncRNA (X26 nt) secreted by GCs can target VE-cadherin to increase angiogenesis and vascular permeability (Chen et al., 2021a). The long-chain non-coding RNA MALAT1 was shown to enhance the expression of classic markers such as VE-cadherin and components of related signaling pathways to promote blood vessel growth and angiogenesis (Li et al., 2017a).

Exosomes are nano-scale membrane vesicles containing proteins, lipids, mRNA, and miRNA that are important in cell-to-cell communications. Signaling pathways composed of exosomes, miRNAs and target genes can affect tumor angiogenesis. For example, miR-135b delivered by gastric tumor exosomes was found to promote angiogenesis by inhibiting FOXO1 expression in endothelial cells (Bai et al., 2019). Similarly, miR-130a activates GC angiogenesis by targeting C-MYB in vascular endothelial cells (Yang et al., 2018b).

## 3 The Mechanism of Resistance to Chemotherapy or Radiotherapy of ncRNAs for Gastric Cancer

**FIGURE 2:**
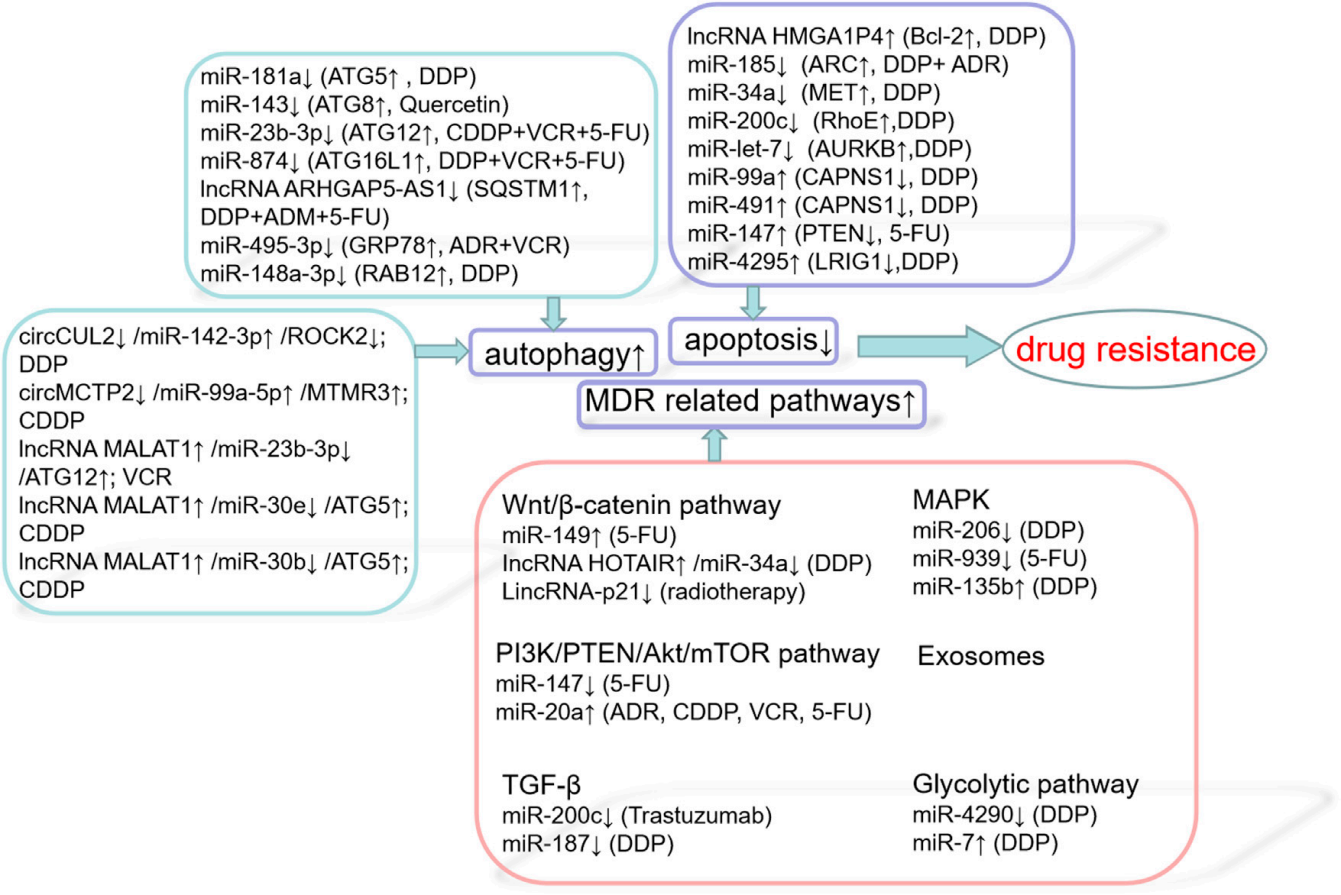
Drug resistance mechanisms of ncRNAs.

The main cause of death in patients with solid tumors is their eventual development of resistance to one or more chemotherapy drugs, which may eventually lead to metastatic disease. The mechanisms involved in GC resistance to cancer drugs are complex, including decreased apoptosis, increased autophagy, lost cell cycle checkpoint control, accelerated cell proliferation, inactivated signaling pathways and targeted genes, as well as accelerated cancer stem cell (CSC) drug metabolism and activation (Figure 2) (Wei et al., 2020a). Chemotherapy is the main treatment for early and late tumors. However, drug resistance is the main obstacle to cancer treatment, which seriously limits the role of traditional chemotherapy and new biological agents (Broxterman et al., 2009). In recent years, there are more and more studies on ncRNAs, target gene regulation, affecting drug function, pharmacogenomics or drug resistance. For example, according to previous studies, the down-regulation of miR-21 makes cancer cells sensitive to different chemotherapy *in vitro*, including cisplatin, etoposide and Adriamycin (Seca et al., 2013; Yang et al., 2015; Vanas et al., 2016). On the other hand, some drugs can induce alterations in miR-21 levels: soladosine can inhibit lung cancer cell invasion through miR-21 down-regulation. miR-34a was reported to be downstream of p53 and to function as a tumor suppressor (Welch et al., 2007). It is down-modulated incolorectal cancer (CRC) (Akao et al., 2010). In 5-Fluorouracil (5-FU)-resistantcolon cancer cells ectopic expression of miR-34a inhibited cell growth and attenuated the resistance to 5-FU through downregulation of SIRT1 and E2F3, inhibition of LDHA and of c-Kit, thus reducing stem cell factor (SCF)-induced migration/invasion (Akao et al., 2011).

### 3.1 Apoptosis

Several ncRNAs affect apoptosis by influencing effective and multifunctional inhibitors of apoptosis, including Bcl-2 (Voss et al., 2010), apoptosis repressor with a caspase recruitment domain (ARC) (Ludwig-Galezowska et al., 2011), MET (Montagne et al., 2015), RhoE (Ongusaha et al., 2006), and AURKB (He et al., 2016); or by influencing enhancers of apoptosis, including calpain small subunit 1 (CAPNS1) (Bertoli et al., 2009), PTEN (Zhu et al., 2020), and LRIG1 (Chang et al., 2013). The lncRNA HMGA1P4 was shown to trigger DDP resistance in GC by adjusting the expression levels of genes associated with apoptosis, including those encoding the proteins Bcl-2, Bax, and caspase-3 (Qiao et al., 2020). Enhanced expression of miR-185 was found to increase the chemosensitivity of GC cells by preventing ARC (Li et al., 2014). Upregulation of miR-34a increased the sensitivity of GC cells to DDP by stopping MET, thereby affecting GC cell proliferation and apoptosis (Zhang et al., 2016b). MiR-200c, which targets RhoE, increases cisplatin-induced apoptosis and is therefore considered a potent factor in eliminating chemoresistance (Ghasabi et al., 2019). MiR-let-7, which targets AURKB, increases the cytotoxicity of DDP in SGC7901/DDP cells by inducing apoptosis and increasing sensitivity to chemotherapeutic agents (Han et al., 2018). Inhibition of miR-99a and miR-491 was found to enhance chemosensitivity to cisplatin in human GC cells by increasing CAPNS1 expression (Zhang et al., 2016c). Down-regulation of miR-147 had a positive effect on PTEN, increasing the sensitivity of GC cells to 5-FU by inducing apoptosis (Shen et al., 2018). MiR-4295 negatively regulates LRIG1 expression to activate the EGFR/PI3K/Akt signal pathway, thereby promoting GC cell proliferation and inhibiting DDP-induced GC cell apoptosis (Yan et al., 2018). Several other ncRNAs affect the apoptosis of GC cells through various pathways. For example, circAKT3, which acts as a ceRNA to miR-198, enhances the expression of *PIK3R1*, activates the PI3K/AKT signaling cascade, and promotes CDDP resistance (Huang et al., 2019). Up-regulating the expression of miR-206, which targets MAPK3 expression, was found to attenuate the proliferation of drug-resistant GC cells, promote apoptosis and reduce DDP resistance (Chen et al., 2019b). Low expression of miR-135b induced the apoptosis of GC cells through the MST1-mediated MAPK signaling pathway, thus enhancing cell sensitivity to cisplatin (Bai et al., 2019).

### 3.2 Autophagy

Autophagy exhibits different effects in different situations. Protective autophagy inhibition reduces previously activated cell defense mechanisms, increasing cell sensitivity to chemotherapeutic agents. Overactivated autophagy can lead to cell death through solute overactivation, activating another cell death pathway, in addition to apoptosis (Taylor et al., 2018). Autophagy is crucial to the mechanism by which GC cells become resistant to chemotherapy. Abnormal activation of autophagy induced by chemotherapeutic drugs can provide energy to support cancer cells, enhancing resistance to chemotherapy. NcRNAs influence autophagy through autophagy-related genes, including *ATG*, *SQSTM1*, *GRP78*, *GABARAPL1*, and *RAB12*. MiR-181a targets *ATG5* as a major autophagy-related modulator and reverses cisplatin resistance in GC cells (Zhao et al., 2016). MiR-143 potently inhibits autophagy by decreasing *GABARAPL1* (*ATG8*), thereby modulating chemosensitivity to quercetin (Du et al., 2015). The miR-23b-3p/ATG12/HMGB2/autophagy regulatory cycle plays a crucial part in multi-drug resistance (MDR) in GC cells. MiR-23b-3p has been shown to inhibit autophagy mediated by *ATG12* and *HMGB2*, sensitizing GC cells to chemotherapy (An et al., 2015). By targeting *ATG16L1*, miR-874 inhibits autophagy and sensitizes GC cells to chemotherapy (Huang et al., 2018). Impaired autophagic degradation of the lncRNA ARHGAP5-AS1 recruited by SQSTM1 in cancer cells resistant to agents such as DDP, ADM, and 5-FU promotes chemoresistance (Zhu et al., 2019a). MiR-495-3p inhibits autophagy and MDR by down-regulating the target gene *GRP78* (Chen et al., 2018a). In addition, miR-148a-3p inhibits cellular protective autophagy in DDP-resistant GC cells by suppressing *RAB12* expression and mTOR1 activation (Li et al., 2017b).

Interactions among ncRNAs also play a crucial role in autophagy in GC. CircCUL2 decreases autophagy through miR-142-3p/ROCK2 to modulate malignant transformation and cisplatin resistance in GC cells (Peng et al., 2020). CircMCTP2 can reduce the autophagy of platinum-resistant GC cells and inhibit the expression of miR-99a-5p and MTMR3, thus inhibiting cisplatin resistance in these. Moreover, inhibition of miR-99a-5p can sensitize GC cells sensitive to CDDP (Sun et al., 2020). The lncRNA MALAT1, an endogenous competitor of miR-23b-3p RNA, attenuates its inhibition of *ATG12* expression, leading to chemotherapy-induced autophagy and resistance to chemotherapy in GC cells (YiRen et al., 2017). The lncRNA MALAT1, which binds to miR-30e to regulate *ATG5* expression, promotes autophagy and inhibits autophagy-related chemotherapy (Zhang et al., 2020c). In addition, the lncRNA MALAT1 has been found to enhance autophagy through the miR-30b/ATG5 axis in HGC-27/CDDP cells, which has a potent effect on autophagy-related CDDP resistance (Xi et al., 2019).

### 3.3 MDR Related Pathways

MDR is the leading cause of chemotherapy failure in cancer treatment. Signaling pathways associated with MDR include the Wnt/β-catenin, PI3K/PTEN/Akt/mTOR, TGF-β, MAPK, and exosome pathways.

#### 3.3.1 Wnt/β-Catenin Pathway

The Wnt/β-catenin pathway is one of the major signaling pathways involved in epithelial-neutral transition (EMT). The EMT-like morphology of cancer cells may be responsible for their chemoresistance and invasion (Chen et al., 2011). MiR-149 promotes 5-FU resistance in GC cells, mainly by targeting *TREM2* and activating the β-catenin pathway (Wang et al., 2020a). Knockdown of the lncRNA HOTAIR and upregulation of miR-34a inhibits DDP resistance in GC cells by inactivating the PI3K/Akt and Wnt/β-catenin signal pathways (Cheng et al., 2018a). LincRNA-p21 enhances the sensitivity of GC to radiotherapy by suppressing the β-catenin signaling pathway (Chen et al., 2019c).

#### 3.3.2 PI3K/PTEN/Akt/mTOR Pathway

The PI3K/PTEN/Akt/mTOR signaling pathway plays a prominent part in mediating drug resistance. Poor outcomes in patients with many types of malignancy has been associated with the loss of *PTEN*, resulting in resistance to new chemotherapeutic agents (Keniry and Parsons, 2008). MiR-147 was found to inhibit the PI3K/AKT signaling pathway by directly increasing *PTEN* expression and enhancing the resistance of GC cells to 5-FU. The miR-20a/LRIG1 axis regulates GC cells through EGFR mediated PI3K/AKT and MAPK/ERK signaling pathways to modulate MDR in GC (Cheng et al., 2018a).

#### 3.3.3 TGF-β

In normal cells and the early stages of cancer, this pathway has tumor-inhibiting functions, including cell cycle arrest and apoptosis. In advanced cancers, however, activation of this pathway can promote tumorigenesis, for example by promoting tumor metastasis (Colak and Ten Dijke, 2017). The TGF-β/ZEB2 axis plays an important role in drug resistance of GC, whereas miR-200C overexpression inhibits ZEB1/ZEB2, leading to sensitizing GC cells to trastuzumab (Zhou et al., 2018). MiR-187 enhances the sensitivity of GC cells to cisplatin by inhibiting the transforming growth factor-β(TGF-β)/Smad signaling pathway (Zhu et al., 2019b).

#### 3.3.4 MAPK

Triple root-activated protein kinase 3 (MAPK3) plays a key role in the extracellular signal-regulated kinase (ERK)/MAPK pathway. Upregulation of miR-206 can inhibit the proliferation of drug-resistant GC cells, promote apoptosis and reduce DDP resistance by targeting *MAPK3* expression (Chen et al., 2019b). Down-regulation of miR-135b leads to inactivation of the MAPK signaling pathway and increases the expression of *MST1* and *Bax*, thus enhancing the sensitivity of GC cells to CDDP. Mir-939 inhibits the growth of GC cells, both *in vitro* and *in vivo*, primarily by inhibiting the activated SLC34A2/Raf/MEK/ERK pathway, and enhances GC cell sensitivity to 5-FU by inducing apoptosis (Zhang et al., 2017b).

#### 3.3.5 Exosomes

Exosomes are biologically active nanosized extracellular vesicles that are released by cells into the extracellular space, and play a central role in the initiation and development of intercellular signaling networks (Rajagopal and Harikumar, 2018). Exosomes can also enhance resistance to chemotherapy by exporting drugs or sharing antiapoptotic drugs in cancer cells, thereby interfering with drug metabolism (Abak et al., 2018).

Cancer-associated fibroblasts (CAFs) support tumor progression and drug resistance by secreting various bioactive substances, including exosomes. MiR-522 secreted by CAFs inhibits iron-associated death in GC and ultimately reduces their sensitivity to chemotherapy (Zhang et al., 2020d). MiR-21-containing exosomes secreted by tumor-associated macrophages (TAMs) induce DDP resistance in GC (Zheng et al., 2017). MGC803/DDP-derived exosomes deliver miR-500a-3p targeting FBXW7 *in vitro*, enhancing DDP resistance and stem cell properties of MGC803 recipient cells (Lin et al., 2020). MiR-155-5p is enriched in MGC-803R exons and can be delivered to MGC-803S cells (Wang et al., 2019). Exosomes containing miR-106a-5p and miR-421 are highly expressed and modulate *TFAP2E* methylation-induced chemotherapy (Jingyue et al., 2019). Exosomes containing miR-501 exhibit resistance to doxorubicin by targeting *BLID* (Liu et al., 2019b). Exosomes secrete nanoparticles with anti-miR-214 activity to reverse GC cell chemoresistance (Wang et al., 2018c). Exosomes containing circPRRX1 enhance doxorubicin resistance by regulating miR-3064-5p/PTPN14 signaling (Table 2) (Wang et al., 2020b).

**TABLE 2:** Exsomes ncRNAs AND drug resistance.

NcRNA	Host cells	Mechanism of function	References
miR-522	Cancer-associated fibroblasts (CAFs)	ferroptosis↓;lipid-ROS accumulation↓	Zhang et al. (2020d)
miR-21	tumor-associated macrophages (TAMs)	exosomal transfer;PTEN↓	Zheng et al. (2017)
miR-500a-3p	cisplatin-resistant GC cells	stemness properties↑	Lin et al. (2020)
miR-155-5p	paclitaxel-resistant GC cells	EMT↑;chemoresistant phenotypes↑	Wang et al. (2019)
miR-106a-5p and miR-421	5-fluorouracil-resistant GC cells	TFAP2E methylation↑	Jingyue et al. (2019)
miR-501	doxorubicin-resistant GC cells	BH3-like motif-containing protein(BLID)↑	Liu et al. (2019b)
miR-214	HEK293T	potential targets↑;apoptosis↑	Wang et al. (2018c)
circPRRX1	doxorubicin-resistant GC cells	miR-3064-5p↓;PTPN14↑	Wang et al. (2020b)

#### 3.3.6 Glycolytic Pathway

Cancer cells undergo glycolysis in the presence of oxygen, which is called the Warburg effect. Accumulated evidence has displayed that the aberrant activation of glycolysis plays an important role in many kinds of diseases via various mechanisms, including the induction of cancer chemotherapy resistance, including GC (Broxterman et al., 2009; Ganapathy-Kanniappan and Geschwind, 2013). A study has determined that microRNA-4290 suppresses PDK1-mediated glycolysis to enhance the sensitivity of gastric cancer cell to cisplatin (Qian et al., 2020). Another research has studied that down-regulation of miR-7 in gastric cancer is able to inhibit the proliferation, colony formation, and glycolysis of GC cells owing to its regulation of LDH-A, it also associated with chemoresistance to cisplatin (Jin et al., 2020a).

### 3.4 The Resistance Mechanism of ncRNAs in HER2-Positive GC

*HER2* overexpression drives tumorigenesis by creating spontaneous receptor homoplasms or heterogenes with other *ERBB* family members, thereby generating the expression of active pathogenic downstream signals, such as PI3K/Akt/mTOR and MAPK, which promote cell proliferation, survival, and angiogenesis (Meric-Bernstam et al., 2019). Trastuzumab, gefitinib, and lapatinib were all shown to have significant curative effects in patients with HER2-positive GC, and these agents have become the key chemotherapy drugs in HER2-positive GC. Silencing of both *HER2* and *EGFR* has been shown to increase tumor chemosensitivity to gefitinib (Wang et al., 2018d).

In HER2-positive GC, miR-494 inhibits cancer-initiating cell phenotypes and reverses resistance to lapatinib by reducing the expression of fibroblast growth factor receptor 2 (FGFR2) (Yu et al., 2018). MiR-143 inhibits the growth of HER2-positive GC cells by inhibiting the KRAS network, including the RNA helicase DDX6 (Tokumaru et al., 2019). The miR-21/PTEN pathway affects the sensitivity of GC cells to trastuzumab by regulating the apoptosis of HER2-positive GC cells (Eto et al., 2014). Overexpression of miR-223 reduced the expression of *FBXW7* and the sensitivity of GC cells to terrazumab, whereas inhibition of miR-223 restored the expression of *FBXW7* and the sensitivity of GC cells to terrazumab (Table 3) (Eto et al., 2015).

**TABLE 3:** NcRNAs in HER2-positive GC.

ncRNA	Main contents	Mechanism of function	References
miR-494	lapatinib	fibroblast growth factor receptor 2(FGFR2)↓;cancer initiating cells (CICs)↓	Yu et al. (2018)
miR-143	growth	KRAS network↓;DDX6 RNA helicase↓	Tokumaru et al. (2019)
miR-21	trastuzumab	PTEN↓;AKT phosphorylation↑	Eto et al. (2014)
miR-223	terrazumab	F-box and WD repeat domain-containing 7 (FBXW7)↓	Eto et al. (2015)

### 3.5 Hypoxia

In addition, ncRNAs affect the sensitivity of GC to chemotherapy by regulating the hypoxia signal pathway. Hypoxia-inducible factor-1 (HIF-1) is the main transcription factor significantly activated by hypoxia (Liu et al., 2008). HIF-1 can inhibit drug-induced apoptosis and reduce the accumulation of drugs in cells, resulting in hypoxia-induced drug resistance. The abnormal expression of miR-20b, miR-27a, and miR-181a is related to the modulation of the chemotherapy response in GC by HIF-1α (Danza et al., 2016). HIF-1α induces the expression of miR-27a, which is closely associated with MDR in GC (Zhao et al., 2015). By binding to miR-376a, the lncRNA Nutm2a-AS1 positively modulates GC formation and drug resistance through the regulation of HIF-1α (Wang et al., 2020c).

## 4 The Effect of Some Important Non-Coding RNAs on Gastric Cancer

**FIGURE 3:**
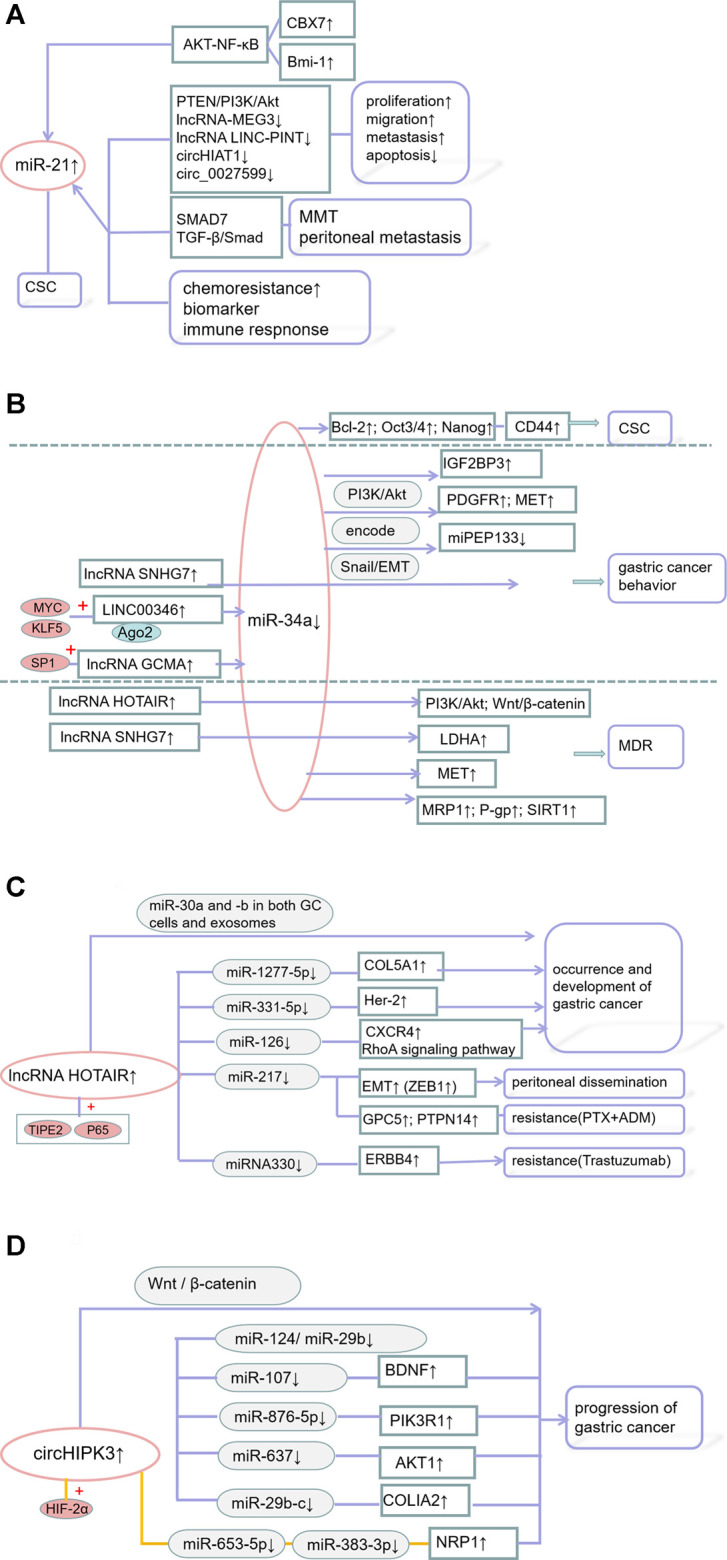
**(A)** The mechanism of miR-21 in GC; **(B)** The mechanism of miR-34a in GC; **(C)** The mechanism of lncRNA HOTAIR in GC; **(D)** The mechanism of circular RNA circHIPK3 in GC.

Comprehensive and in-depth analyses of several ncRNAs have revealed their roles in GC cell behavior and drug sensitivity (Figure 3).

### 4.1 MiR-21

For example, a large-scale miRnome analysis of 540 samples, including solid tumors, such as GCs found that miR-21 is closely associated with the pathogenesis of solid tumors and poor patient prognosis (Volinia et al., 2006). MiR-21 affects the proliferation, metastasis, apoptosis, stem cell properties, and drug sensitivity of GCs. For example, miR-21-5p provides a favorable *in vitro* environment for the mesothelial-to-mesenchymal transformation (MMT) of peritoneal metastatic cancer cells by activating the TGF-β/Smad pathway (Li et al., 2018b). Chromosomal protein homology (CBX) maintains the stem cell-like properties of GC cells by regulating the p16 and AKT-NF-κB-miR-21 pathways (Ni et al., 2018). B cell-specific moloney murine leukemia virus integration site 1 (Bmi-1) actively regulates the stem cell-like properties of GC cells by increasing miR-21, which targets the PTEN/Akt signaling pathway and promotes human GC cell proliferation, migration, and apoptosis (Zhang et al., 2012; Wang et al., 2016; Wang et al., 2018e). The lncRNAs-MEG3 (Dan et al., 2018) and LINC-PINT (Feng et al., 2019), as well as circHIAT1 (Quan et al., 2020) and circ_0027599 (Han et al., 2021), are able to bind miR-21 and play a negative role in GC progression. Exosomes carrying miR-21 can be transferred from macrophages to GC cells, inhibiting cell apoptosis, enhancing the activation of the PI3K/AKT signaling pathway, and inducing cisplatin resistance by down-regulating *PTEN*. MiR-21 enhances cell survival by targeting *PTEN*, inducing resistance to doxorubicin and cisplatin (Yang et al., 2013; Chen et al., 2018b).

Serum miR-21 is also a biomarker and a key regulator of the immune response in GC. Serum miR-21 concentration can indicate tumor recurrence in young GC patients (Park et al., 2016). MiR-21 is not only highly expressed in GCs, but is also highly expressed in the gastric juice of GC patients, affecting their prognosis (Motoyama et al., 2010; Zheng et al., 2011; Cui et al., 2013; Kim et al., 2013; Wu et al., 2015; Sierzega et al., 2017; Pereira et al., 2019; Zhang et al., 2020e). MiR-21 may also be a potential indictor of chemoresistance patients with metastatic GC (MGC) (Zheng et al., 2019). The overexpression of miR-21 increases the percentage of Th17 cells and reduces the percentage of Treg cells, resulting in an imbalance in the PD-1/PD-L1 pathway and regulating immune responses in GC (Liu et al., 2018). Synthetic circRNAs that target miR-21 can induce therapeutic dysfunction, inhibiting the proliferation of cancer cells and the activity of miR-21 against downstream protein targets, including the tumor protein DAXX (Qi et al., 2017b).

### 4.2 MiR-34a

MiR-34a was found to be a classic tumor suppressor miRNA in several malignancies (Hermeking, 2007). MiR-34a has also been shown to inhibit the growth, invasion, and metastasis of GC by targeting the expression of PDGFR and MET (Peng et al., 2014). Silencing of miR-34a is also partly responsible for activation of the cancer-promoting molecule IGF2BP3 (Zhou et al., 2017b). The novel microprotein miPEP133 encoded by the miR-34a precursor enhances *p53* transcription and miR-34a expression, thereby exerting a tumor suppressor effect (Kang et al., 2020). SNHG7 increases the invasion and migration of GC cells through the miR-34a-Snail-EMT axis (Zhang et al., 2020f). LINC00346 binds to and neutralizes miR-34a-5p, with KLF5 and MYC/LINC00346/miR-34a-5p being key effectors of GC tumorigenesis and progression (Xu et al., 2019a). SP1-activated lncRNA GCMA is a ceRNA that adsorbs miR-124 enhancing tumor metastasis and reducing GC progression (Tian et al., 2020b). Bmi-1 can actively regulate the stem cell-like properties of GC cells by increasing miR-34a expression (Wang et al., 2016). MiR-34a can regulate the sensitivity of human GC cells to DDP by targeting MET (Zhang et al., 2016b). MiR-34a-5p regulates the expression of Sirtuin 1 (SIRT1), P-glycoprotein (P-gp), and multidrug resistance-related protein 1 (MRP1) by directly binding to the 3′untranslated region (UTR) of *SIRT1*, thereby reversing the MDR of GC cells (Deng et al., 2021).

The lncRNAs HOTIAR and SNHG7 adsorb miR-34a. Anti-miR-34a antibody was found to reverse the effect of si-HOTAIR on DDP resistance, on apoptosis-related genes, and on the PI3K/Akt, and Wnt/β-catenin signaling pathways in anti-DDPGC cells, suggesting that the effect of HOTAIR depends on miR-34a (Cheng et al., 2018a). The level of the lncRNA SNHG7 negatively correlates with that of miR-34a, desensitizing GC cells to cisplatin (Pei et al., 2021). In addition, miR-34a was found to affect the expression of cancer stem cells (CSCs) overexpressing CD44, leading to tumorigenesis and recurrence, while inhibiting the proliferation, metastasis, and survival of CD44-positive CSCs (Jang et al., 2016).

### 4.3 LncRNA HOTAIR

Overexpression of the lncRNA HOTAIR is a biomarker for poor prognosis in patients with GC, as it may enhance malignant phenotype (Feng and Huang, 2017). High expression of HOTAIR is associated with tumor differentiation, lymph node and distant metastases, and higher clinical stage (Xu et al., 2019b). HOTAIR promotes GC by altering miRNA levels in cells and exosomes (Zhang et al., 2020g). HOTAIR effectively binds to and neutralizes miR-331-3p, thereby regulating the attenuation of HER2 levels and promoting GC progression (Liu et al., 2014). HOTAIR enhances GC growth by binding to a neutralizing miR-1277-5p and by up-regulating COL5A1 (Wei et al., 2020b). High HOTAIR expression promotes the proliferation and metastasis of GC through the miR-126/CXCR4 axis and SDF-1/CXCR4 signaling (Xiao et al., 2019). In mice, HOTAIR directly targets miR-217 and combines with the zinc finger electronic box binding home box 1 protein (ZEB1) to inhibit peritoneal diffusion of GC, significantly prolonging survival time (Takei et al., 2020). HOTAIR, which is stimulated by the NF-κB pathway, was found to promote GC progression by enhancing non-resolving inflammation (Zhang et al., 2019d). HOTIAR also affects GC chemoresistance. Overexpression of HOTAIR was found to enhance the resistance of GC cells to paclitaxel (PTX) and doxorubicin (ADR) (Wang et al., 2018f). In addition, the HOTAIR-miR-330-ERBB4 regulatory network with miRNA330 as its core was found to enhance the sensitivity of tumor cells to trastuzumab (Bie et al., 2020).

### 4.4 Circular RNA circHIPK3

CircHIPK3 is generated from exon 2 of the gene encoding homeodomain-interacting protein kinase 3 (HIPK3) (Xie et al., 2020b; Wen et al., 2020). The expression of circHIPK3 was found to be significantly higher in GC tissues than in adjacent normal tissues, suggesting that increased CircHIPK3 expression was associated with poor prognosis (Liu and Xu, 2019). CircHIPK3 forms an axis with miR-124 and miR-29b to that target COL1A1, COL4A1, and CDK6, which function in different histological growth patterns (Cheng et al., 2018b). CircHIPK3 adsorbs miR-107 (Wei et al., 2020c), miR-876-5p (Li et al., 2020c), miR-637 (Yang et al., 2021), playing a pivotal role in GC tumorigenesis and development. Hif-2α is upregulated in hypoxic drug-resistant GC (HRGC) cells under a long-term hypoxic microenvironment and promotes GC metastasis through the miR-653-5p/miR-338-3P-NRP1 axis (Jin et al., 2020b). These circRNAs, which form complex RBP-circRNA-miRNA-mRNA interaction networks, such as the circHIPK3/miR-29b-c/COL1A2 network, are involved in GC development, progression, and reduced sensitivity to chemotherapy (Pereira et al., 2020).

## 5 Clinical Trials of ncRNAs

Clinical studies are underway to evaluate applications of ncRNAs. For example, a study of tumor and adjacent normal tissues collected from 60 prospectively selected patients with GC found that Hsa_circ_0000745 played a crucial role in tumor development. A diagnostic index, involving plasma concentrations of ncRNAs and CEA, has been shown promising in the identifying patients with malignant tumors (Huang et al., 2017b). Four common miRNA polymorphisms, miR-146aC > G, miR-149T > C, miR-196a2T > C, and miR-499A > G, were found to be associated with susceptibility to and prognosis of GC in the Korean population (Ahn et al., 2013). A prospective clinical trial of GC patients treated with oxaliplatin/capecitabine (XELOX) chemotherapy found that plasma concentrations of miR-17-92 were closely associated with the progression of advanced GC and the effectiveness of XELOX chemotherapy (Fan et al., 2018). A study in 2010–2017 of Pralatrex and oxaliplatin in the treatment of unresectable or metastatic esophageal, gastric, or gastroesophageal junction carcinoma compared the mean expression of miR-215-5p in tumor tissues of responders and nonresponders using microprocessing devices of a gene chip and obtained reliable results. Another study in Singapore aims to test the predictive ability of multiple blood biomarkers, such as miRNAs, to detect early signs of disease at a stage at which tumors can be prevented or cured (Song and Meltzer, 2012).

Ongoing studies are evaluating the mechanisms of action of lncRNAs in gastrointestinal diseases and their possible relationships with *Helicobacter pylori* infection. In addition, levels of expression of miRNAs in GC tissue and blood and their association with responses to chemotherapy are being determined by next-generation sequencing, with validation by qRT-PCR, in multiple independent patient cohorts. The relationships between ncRNAs and cancer immune checkpoints are being explored to identify new uses for ncRNAs in cancer immunotherapy, and differences in exosomal protein/ncRNA components are being evaluated in patients before and after combination chemotherapy with Apatinib and anti-PD-1 antibody.

## 6 Discussion

Findings to date have shown that ncRNAs are extensively involved in gene regulatory networks. Many ncRNAs do not act as a single link, but as branching points with broad outputs that affect regulatory networks containing target genes or related signaling pathways. In GCs, ncRNAs have been identified as carcinogenic drivers and tumor suppressors.

In conclusion, this review provides strong evidence that ncRNAs can act as biomarkers for the diagnosis, prognosis, and chemotherapy resistance of GCs. Many studies to date have shown that ncRNAs play complex and important roles in GC proliferation, invasion, apoptosis, and angiogenesis. These ncRNAs or their resulting ceRNAs can alter the expression of target genes and/or affect classic signaling pathways. ncRNAs also participate in the regulation of resistance to radiotherapy and chemotherapy by affecting the expression of signaling pathways related to apoptosis and autophagy, and by regulating MDR-related genes and pathways. ncRNAs can also form networks in GC. Taken together, these findings indicate that targeting ncRNAs may be a promising method of enhancing sensitivity to chemotherapy, thereby improving the efficacy of treatment, in patients with GC. Studies evaluating the effects of ncRNAs on immunotherapy in GC are currently ongoing. The stability of ncRNAs in the circulation makes them suitable diagnostic and prognostic markers for most cancers and determination of their concentrations in serum may be useful in the personalized management of patients. Better understanding of these ncRNAs and their target genes may provide new perspectives for the development of more complex and effective therapeutic agents for the treatment of GC. Techniques and tools to develop ncRNA-targeted and ncRNA-based drugs have been widely used in cancer treatment. Strategies including antisense oligonucleotide (ASO), RNA interference (RNAi) and CRISPR/Cas9 have been proposed to up-regulate the tumor inhibition of ncRNA using gene silencing techniques (Chen et al., 2021b). However, barriers to the translation of nucleic acid-based therapeutics into the clinic are related to their stability, specificity, delivery, and toxicity issues, such as “on-target” and “off-target” side effects (Hueso et al., 2021). It is very important to conduct further *in vitro* and *in vivo* studies and clinical studies on the efficacy and safety of ncRNA to achieve precision medicine.
